# Asymptomatic Portal Vein Aneurysm Uncovered During the Evaluation of a Gastrointestinal Hemorrhage: A Rare Clinical Case

**DOI:** 10.7759/cureus.68388

**Published:** 2024-09-01

**Authors:** Hui Un Kim, Heather L Mateja, Ruben Neris, Ali Kimyaghalam, Peter M DeVito

**Affiliations:** 1 General Surgery, Western Reserve Health Education/NEOMED, Warren, USA; 2 General Surgery, American University of Antigua, Osbourn, ATG; 3 Vascular Surgery, Western Reserve Health Education/NEOMED, Warren, USA

**Keywords:** vascular anomaly, portal hypertension, acquired aneurysm, congenital aneurysm, gastrointestinal bleeding, portal vein aneurysm

## Abstract

Portal vein aneurysms (PVAs) are rare vascular anomalies that are most often discovered incidentally during imaging for unrelated conditions. Their management remains controversial due to the limited data available. Here, we report the case of a 72-year-old male who presented with gastrointestinal bleeding and was found to have an incidental 4.9 cm PVA on abdominal computed tomography angiography (CTA), managed conservatively with regular follow-up imaging and monitoring. PVAs, while rare, present a significant clinical challenge due to the lack of consensus on their management. Treatment strategies range from conservative monitoring to surgical intervention, depending on the presence of symptoms, aneurysm size, and underlying etiology. This case highlights the importance of a multidisciplinary approach in managing PVAs, particularly when they are discovered incidentally. Further research is needed to develop standardized guidelines that address the nuances of treating both congenital and acquired PVAs.

## Introduction

The portal vein can be distinguished by the presence of capillaries on both ends and the absence of valves unlike other veins in the body [1]. Aneurysms here are very rare, representing only 3% of all venous aneurysms [2,3]. The first known case of a portal vein aneurysm (PVA) was described in 1956 after the rupture of a hemocholecyst secondary to this aneurysm [3,4]. However, we still do not fully understand how it develops [2]. There has been an established relationship between chronic liver disease and portal hypertension leading to hypertrophy, weakening of the wall, and aneurysmal dilation [2]. Congenital etiology has also been postulated, with the likely dilatation coming from a remanent of the right primitive vitelline vein [1]. PVAs are most commonly located at the level of the main portal vein (66%) followed by the porto-splenic junction (PSJ) (17%), the splenic-superior mesenteric vein junction (SSMVJ) (11%), and the right portal vein (RPV) (6%) [5].

The maximum diameter of the portal vein has been widely debated, but it is generally accepted that the portal vein diameter should not exceed 19 mm in cirrhotic patients and 15 mm in a normal liver [1-3]. Therefore, an aneurysm larger than 20 mm is considered pathologic [1-3]. Identifying these aneurysms can be challenging due to the variability in symptoms. One study reported that 25-30% of patients were asymptomatic, 30-50% experienced non-specific abdominal pain, and for up to 15% of patients, the symptomology was unclear [2]. However, the symptoms of PVAs may also be related to portal hypertension, varices, or compression of adjacent organs, which further complicate the clinical picture [2,3]. Treatment typically depends on the presence or absence of symptoms, the location and size of the aneurysm, and the patient’s comorbidities, but there are still no clear guidelines on the management for these patients as it is unclear whether the benefits of operative management outweigh the clinical risk of rupture [2,3,5].

## Case presentation

A 72-year-old male with a past medical history significant for hypertension and chronic obstructive pulmonary disease (COPD) presented to the emergency department with a chief complaint of gastrointestinal (GI) bleeding. The patient reported experiencing melena for the past two days and lightheadedness without any associated abdominal pain or vomiting. He denied any recent changes in bowel habits, weight loss, or jaundice.

On initial examination, the patient appeared pale and mildly tachycardic, with a heart rate of 105 beats per minute. Blood pressure was 135/85 mmHg. His abdomen was soft, non-tender, and non-distended, with no palpable masses or organomegaly. Rectal examination confirmed the presence of melena. Laboratory tests revealed a hemoglobin level of 8.5 g/dL (a significant drop from his baseline of 13.0 g/dL), elevated blood urea nitrogen (BUN) at 35 mg/dL, and a normal liver function test. Coagulation studies were within normal limits.

**Figure 1:**
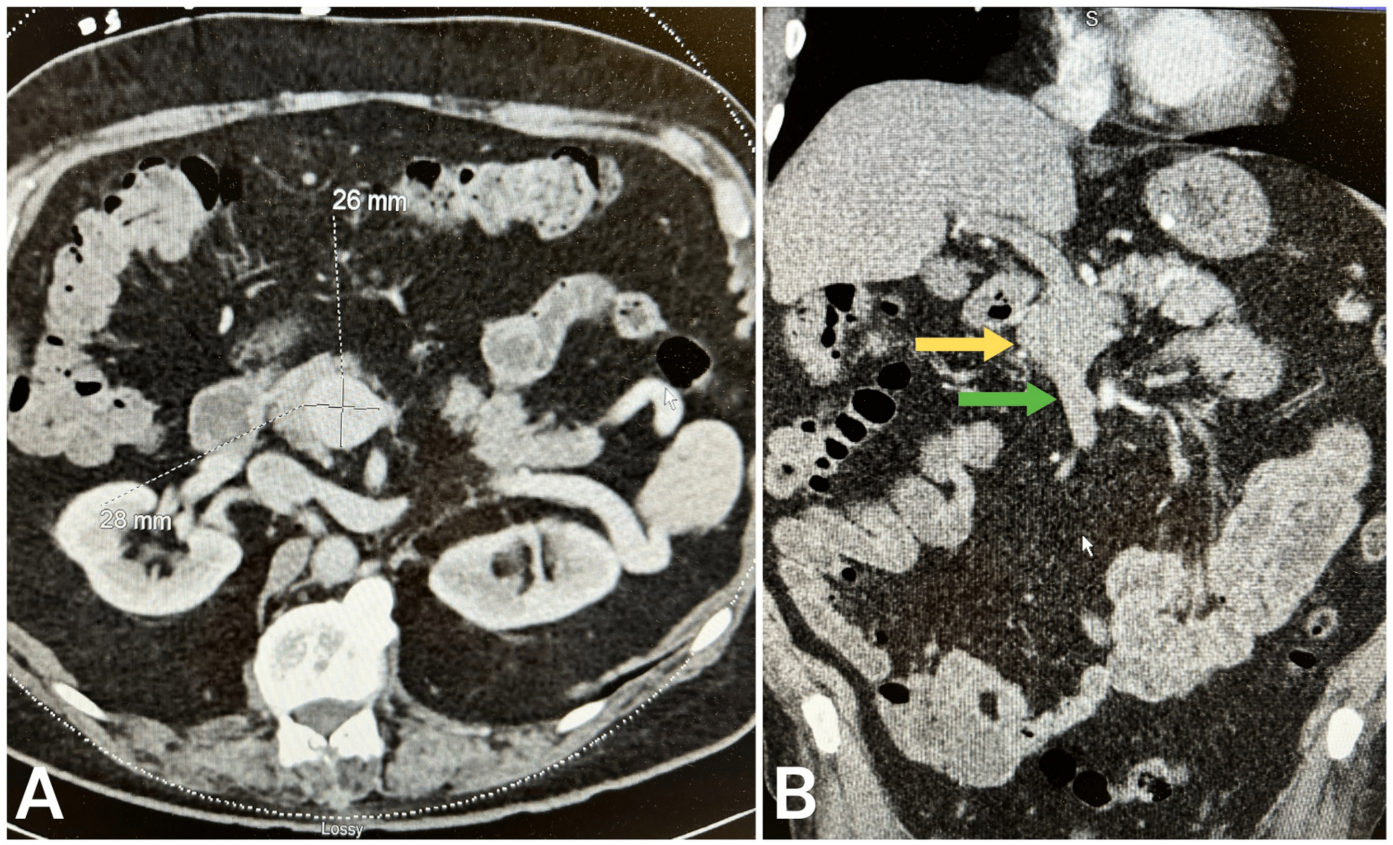
Abdominal computed tomography angiography (CTA) showing a portal vein aneurysm (yellow arrow) above the confluence (green arrow) measuring up to 2.8 x 2.6 x 4.9 cm. (a) Axial view. (b) Coronal view.

While awaiting endoscopic evaluation, the patient underwent an abdominal computed tomography angiography (CTA) to assess the patient’s vasculature and rule out any potential intraabdominal pathology. The abdominal CTA incidentally revealed a 2.8 x 2.6 x 4.9 cm aneurysm of the portal vein (Figure 1) without any other significant findings. The aneurysm was located at the main portal vein above the confluence of the splenic and superior mesenteric veins. There was no evidence of thrombosis or rupture. 

Given the incidental finding of the PVA, vascular surgery was consulted. The patient was asymptomatic from the aneurysm, and there were no signs of complications. After discussion, it was decided to manage the PVA conservatively with regular follow-up imaging and monitoring of liver function. The patient was advised on the importance of follow-up and was scheduled for a repeat CTA in six months to assess for any changes in its size. Upper endoscopy revealed the GI bleeding was from a duodenal ulcer, which was subsequently resolved via endoscopic cauterization and medical management.

## Discussion

PVAs are rare vascular anomalies. Two publications have recognized fewer than 300 cases in the literature as of 2023 [2,3]. The management of PVAs remains controversial due to the scarcity of data, and there is no clear consensus on the best treatment strategies, which vary significantly based on the clinical presentation, size, location, and etiology of the aneurysm [3,5,6].

The management of PVAs can be broadly categorized into conservative, interventional, and surgical approaches [3,5,6]. Conservative management, including regular monitoring with imaging studies such as ultrasound, is often recommended for asymptomatic PVAs, especially those detected incidentally and not associated with complications such as thrombosis, rupture, or compression of adjacent structures such as in our patient [5,7]. Ahmed et al. (2021) reported that non-operative management was safe and feasible in most cases, with favorable long-term outcomes [5]. However, the decision to proceed with conservative management should be individualized, considering factors such as aneurysm size, growth rate, and the presence of symptoms [2].

For symptomatic PVAs, or those with a high risk of complications, surgical intervention may be warranted [6]. Surgical options include aneurysm resection, splenectomy, or portosystemic shunting, depending on the location and extent of the aneurysm [6,8]. Fleming et al. (2005) noted that operative intervention might be necessary for extrahepatic portomesenteric venous aneurysms with associated complications, although the outcomes of such surgeries require careful consideration [6]. In addition, endovascular approaches, such as coil embolization or stent placement, have been explored in select cases, particularly when surgical risks are high [2,3,6]. Utilizing different imaging modalities can aid in deciding the appropriate treatment strategy. Sonography, contrast-enhanced CT, magnetic resonance imaging (MRI), endoscopic ultrasound, and intraductal ultrasound have all been described as possible modalities to identify and monitor PVAs [2].

The distinction between congenital and acquired PVAs is important in determining the appropriate management strategy. Congenital PVAs are often associated with developmental anomalies of the portal venous system and may present in younger patients [9]. They are generally asymptomatic and have a lower risk of complications, making conservative management the preferred approach [9]. Burdall et al. (2016) highlighted the association of congenital PVAs with other vascular malformations, particularly in patients with genetic conditions, such as trisomy 21 [9]. In these cases, routine surveillance with imaging is typically sufficient unless the aneurysm exhibits rapid growth or becomes symptomatic [2,3].

On the other hand, acquired PVAs, which develop later in life, are often related to underlying conditions such as liver cirrhosis, portal hypertension, or trauma [1,2,7]. These aneurysms may carry a higher risk of rupture or thrombosis, particularly in the presence of cirrhosis or portal hypertension, necessitating a more aggressive management approach [3,6]. In cases of acquired PVAs secondary to portal hypertension, addressing the underlying cause, such as controlling portal pressure, may be an essential component of the treatment strategy [2,5]. Prior imaging for various unrelated complaints did not demonstrate a PVA in our patient, making congenital an unlikely etiology, although the patient also has no history of liver disease or portal hypertension, further complicating this incidental finding. Conservative management with regular follow-up imaging should be sufficient in managing our patient’s PVA, although more thorough studies demonstrating long-term outcomes are needed to fully elucidate proper guidelines.

## Conclusions

The management of PVAs remains a challenge due to the limited data available and the rarity of the condition. Conservative management is generally favored for asymptomatic and congenital PVAs, while symptomatic or high-risk aneurysms may require surgical or interventional treatment. The decision-making process should be multidisciplinary, involving hepatologists, vascular surgeons, and interventional radiologists, to tailor the treatment approach to the individual patient’s clinical context. Further research is needed to establish standardized guidelines for the management of PVAs, particularly in distinguishing the optimal strategies for congenital versus acquired aneurysms.
